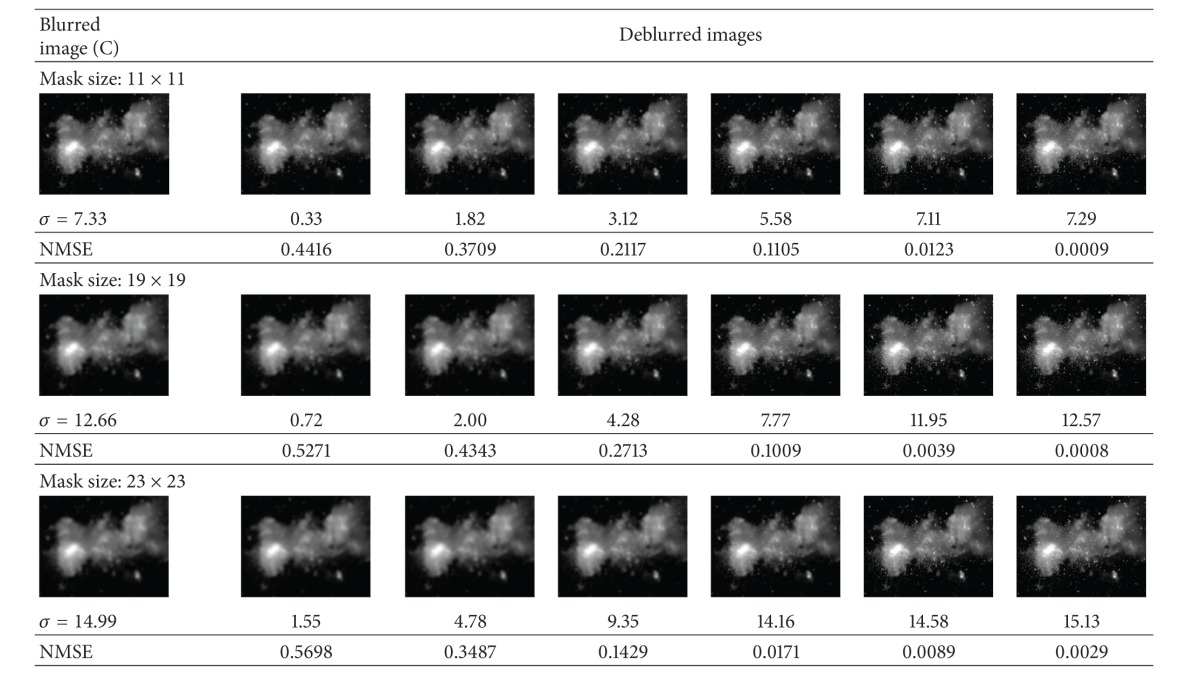# Image Deconvolution by Means of Frequency Blur Invariant Concept

**DOI:** 10.1155/2014/951842

**Published:** 2014-08-12

**Authors:** Barmak Honarvar Shakibaei, Peyman Jahanshahi

**Affiliations:** Integrated Lightwave Research Group, Department of Electrical Engineering, Faculty of Engineering, University of Malaya, 50603 Lembah Pantai, Kuala Lumpur, Malaysia

## Abstract

Different blur invariant descriptors have been proposed so far, which are either in the spatial domain or based on the properties available in the moment domain. In this paper, a frequency framework is proposed to develop blur invariant features that are used to deconvolve a degraded image caused by a Gaussian blur. These descriptors are obtained by establishing an equivalent relationship between the normalized Fourier transforms of the blurred and original images, both normalized by their respective fixed frequencies set to one. Advantage of using the proposed invariant descriptors is that it is possible to estimate both the point spread function (PSF) and the original image. The performance of frequency invariants will be demonstrated through experiments. An image deconvolution is done as an additional application to verify the proposed blur invariant features.

## 1. Introduction

Blur is one of the degradations that is classified as radiometric, created by factors such as motion, overexposure, camera vibrations, and strong illumination. The point spread function (PSF) of an imaging system introduces some levels of blurring in the captured images. Mostly the PSF is modeled as a Gaussian distribution which is widely applicable in imaging devices [1]. Restoration of images can be performed using nonblind and blind techniques. In case of nonblind technique [2–4], the estimation of the original image is obtained using prior knowledge of the PSF which can be derived based on the various modeling algorithms [5, 6]. But in most cases, the PSF is unknown. Hence, in order to estimate the PSF and the original image, blind deconvolution technique is adopted [7–13]. The general model which is used for the observed blurred image, *g*(*x*, *y*) of a scene, *f*(*x*, *y*), is described by a convolution integral:

(1) $$\begin{array}{c} g\left(x,y\right)=f\left(x,y\right)⊗h\left(x,y\right)+n\left(x,y\right), \end{array}$$

where *h*(*x*, *y*) and *n*(*x*, *y*) are the PSF kernel and random noise, respectively. Here, ⊗ denotes the 2D linear convolution.

Earlier work in image features invariant with respect to blur is divided into three different categories. First category belongs to deriving blur invariant properties of the PSF. The second one is to estimate the PSF, and the last is to obtain the original image via deblurring processes. A frequency domain approach for blur invariant has been done to develop some features for object recognition [14]. Blur invariant in moment domain is proposed by Flusser and Suk,which is invariant to convolution of an image with an arbitrary symmetric PSF kernel [15]. Two recognition methods for motion blurred images have been developed based on a relation between the moments of the blurred and the original images [16]. A set of invariants are derived from Zernike moments which are simultaneously invariant to similarity transformation and to convolution with circularly symmetric PSF [17]. Dai et al. [18] proposed a solution to develop a blur invariant feature set for degraded image recognition systems based on the orthogonal Legendre moments. Yan et al. [19] used the second order central moment minimization for restoring of the astronomical images degraded by the atmospheric turbulence. Khan et al. presented a biometric technique for identification of a person using the iris image by means of the ordinary moments and *k*-means algorithm [20]. In this method, the iris is segmented from the acquired image of an eye using an edge detection algorithm. Wavelet-domain blur invariants are proposed for 2D discrete signals which are invariant to centrally symmetric blurs [21]. Honarvar et al. [22] derived new algorithms for image deblurring by means of image reconstruction from its complete set of geometric and complex moments. The adaptive total variation (TV) minimization technique by Yoon et al. [23] has used image enhancement from flash and no-flash pairs. Accurate sparse-projection image reconstruction via nonlocal TV regularization is proposed by Zhang et al. for tomography applications [24].

In this paper, the blur invariant features in Fourier domain are proposed. By establishing a relationship between the normalized Fourier transform of the blurred and the original images, it is possible to estimate the original image.

## 2. Frequency Domain Concerns

In this section, we establish a new frequency blur invariant based on a Gaussian PSF for degraded images. Since an imaging system can be modeled as a 2D convolution in (1), it is possible to transform this equation to the Fourier domain. For frequency analysis, we consider the imaging system in the presence and absence of noise, respectively.

### 2.1. Noise Effect

In the presence of noise, the degradation model can be expressed in the Fourier domain as

(2) $$\begin{array}{c} G\left(u,v\right)=F\left(u,v\right)H\left(u,v\right)+N\left(u,v\right), \end{array}$$

where *G*(*u*, *v*), *F*(*u*, *v*), *H*(*u*, *v*), and *N*(*u*, *v*) are the frequency responses of the observed image, original image, PSF, and noise, respectively. The Wiener deconvolution method has widespread use in image deconvolution applications, as the frequency spectrum of most visual images is fairly well behaved and may be estimated easily [25, 26]. Here, the target is to find *λ*(*x*, *y*) in the way that $\hat{f}\left(x,y\right)$ can be approximated as a convolution, that is, *λ*(*x*, *y*) ⊗ *g*(*x*, *y*), to minimize the mean square error, where $\hat{f}\left(x,y\right)$ is an estimation of *f*(*x*, *y*). The Wiener deconvolution filter provides such a *λ*(*x*, *y*). The filter is described in the frequency domain:

(3) $$\begin{array}{c} \Lambda \left(u,v\right)=\frac{H^{*}\left(u,v\right)S\left(u,v\right)}{{\left|H\left(u,v\right)\right|}^{2}S\left(u,v\right)+N\left(u,v\right)}, \end{array}$$

where *S*(*u*, *v*) is the mean power spectral density (*S*(*u*, *v*) = **E**{|*X*(*u*,*v*)|^2^}) of the original image and *f*(*x*, *y*) and the superscript ∗ denote complex conjugation. Using this technique to find the best reconstruction of a noisy image can be compared with other algorithms such as Gaussian filtering.

### 2.2. Proposed Frequency Blur Invariant

If noise *n*(*x*, *y*) is neglected, (2) can be reduced to

(4) $$\begin{array}{c} G\left(u,v\right)=F\left(u,v\right)H\left(u,v\right). \end{array}$$

Here, we consider a Gaussian distribution for the PSF as

(5) $$\begin{array}{c} h\left(x,y\right)=\frac{1}{2\pi \sigma^{2}}e^{-\left(x^{2}+y^{2}\right)/2\sigma^{2}}. \end{array}$$

Assume that the imaging system does not change the overall brightness of the image; that is,

(6) $$\begin{array}{c} \overset{+\infty }{\underset{-\infty }{\iint }}h\left(x,y\right)dx\;dy=1. \end{array}$$

It is clear that (5) is a separable function in terms of *x* and *y*, and we can rewrite that as follows:

(7) $$\begin{array}{c} h\left(x,y\right)=h\left(x\right)h\left(y\right)=\left(\frac{1}{\sigma \sqrt{2\pi }}e^{-x^{2}/2\sigma^{2}}\right)\left(\frac{1}{\sigma \sqrt{2\pi }}e^{-y^{2}/2\sigma^{2}}\right). \end{array}$$

In order to obtain the Fourier transform of the 2D Gaussian PSF, it is easy to consider the 1D PSF, and using the formula in [27], we have

(8) $$\begin{array}{c} h\left(x\right)=\frac{1}{\sigma \sqrt{2\pi }}e^{-x^{2}/2\sigma^{2}}\overset{F}{⟷}H\left(u\right)=e^{-\sigma^{2}u^{2}/2}. \end{array}$$

For a 2D PSF, because of its separability property, the Fourier transform of (7) can be written as

(9) $$\begin{array}{c} H\left(u,v\right)=e^{-\sigma^{2}(u^{2}+v^{2})/2}. \end{array}$$

Substituting (9) into (4), we get

(10) $$\begin{array}{c} G\left(u,v\right)=F\left(u,v\right)\cdot e^{-\sigma^{2}(u^{2}+v^{2})/2}. \end{array}$$

To obtain the frequency domain blur invariant, we set both frequencies (*u*, *v*) to (1,1) in (10) which leads to

(11) $$\begin{array}{c} e^{-\sigma^{2}}=\frac{G\left(1,1\right)}{F\left(1,1\right)}. \end{array}$$

The PSF kernel *σ* can be eliminated by substituting (11) into (10) which yields

(12) $$\begin{array}{c} \frac{G\left(u,v\right)}{{\left[G(1,1)\right]}^{{(u}^{2}+v^{2})/2}}=\frac{F\left(u,v\right)}{{\left[F(1,1)\right]}^{\left(u^{2}+v^{2}\right)/2}}. \end{array}$$

Equation (12) shows the proposed blur invariant features in Fourier domain for all range of frequencies which is independent of the Gaussian blur kernel (*σ*). In this paper, these features are obtained by normalizing the Fourier transform of the original and blurred images with their respective Fourier transforms, *F*(1,1) and *G*(1,1).

## 3. Image Deconvolution

In this section, we show an image deconvolution method based on the derivatives of the blurred image function which are defined in terms of differences. We begin with 1D version of (10) because the separable property of the Gaussian distributions will allow the easy 2D implementation of them. To deconvolve the original signal, we rewrite (10) in 1D form as

(13) $$\begin{array}{c} F\left(u\right)=e^{\sigma^{2}u^{2}/2}G\left(u\right). \end{array}$$

Since the inverse Fourier transform of the function *e* ^*σ*^2^*u*^2^/2^ does not exist, we can not find an explicit form of that to find the original signal deconvolution from (13). If we use the Taylor series expansion of the squared exponential function, it is possible to connect the degraded signal to its original form. Equation (13) leads to

(14) $$\begin{array}{c} F\left(u\right)=\left(1+\frac{\sigma^{2}u^{2}}{2}+\frac{\sigma^{4}u^{4}}{8}+\frac{\sigma^{6}u^{6}}{48}+\cdots \right)G\left(u\right). \end{array}$$

By using the high order derivative property of the Fourier transform (differentiation property), we have

(15) $$\begin{array}{c} \frac{d^{2k}g\left(x\right)}{dx^{2k}}\overset{F}{⟷}{\left(ju\right)}^{2k}G\left(u\right). \end{array}$$

Using (15) in (14) and taking the inverse Fourier transform yields

(16) $$\begin{array}{c} f\left(x\right)=\overset{\infty }{\underset{k=0}{\sum }}\frac{1}{k!}{\left(\frac{-\sigma^{2}}{2}\right)}^{k}\frac{\partial^{2k}g\left(x\right)}{\partial x^{2k}}. \end{array}$$

Equation (16) includes the even order derivative of the degraded signal that can be defined as a difference. For example, the definition of a second-order derivative as the difference is [28]

(17) $$\begin{array}{c} \frac{\partial^{2}g\left(x\right)}{\partial x^{2}}=g\left(x+1\right)+g\left(x-1\right)-2g\left(x\right). \end{array}$$

By generalizing the definition of a high order derivative as differences, we are able to approximate the continuous derivatives with discrete differences as

(18) $$\begin{array}{c} \frac{\partial^{2k}g\left(x\right)}{\partial x^{2k}}=\overset{2k}{\underset{m=0}{\sum }}{\left(-1\right)}^{2k-m}\left(\begin{array}{c} 2k\\ m \end{array}\right)g\left(x-k+m\right). \end{array}$$

Sustituting (18) into (16) yields the original signal in terms of the degraded signal as

(19) $$\begin{array}{c} f\left(x\right)=\overset{\infty }{\underset{k=0}{\sum }}\frac{1}{k!}{\left(\frac{-\sigma^{2}}{2}\right)}^{k}\overset{2k}{\underset{m=0}{\sum }}{\left(-1\right)}^{2k-m}\left(\begin{array}{c} 2k\\ m \end{array}\right)g\left(x-k+m\right). \end{array}$$

Similarly, for a 2D blurred image, the desired image deconvolution can be obtained from

(20) f(x,y)=∑k=0∞∑l=0∞1k!l!(−σ22)k+l∑m=02k∑n=02l(−1)2k+2l−m−n  ×(2ki)(2lj)g(x−k+m,y−l+n).

## 4. Experimental Studies

Different numerical experiments are conducted in order to prove the validity and the efficiency of the proposed methods. The detailed description of these numerical experiments will be presented in this section. The performance for the proposed methods is evaluated based on the binary, gray-scale, and real images. This section is divided into two subsections.

In the first subsection, the accuracy of the proposed blur invariant features in the Fourier domain is validated by using the frequency analysis of the blurred and the original images. The efficiency of the proposed image deconvolution algorithm based on the Gaussian PSF is carried out with different experiments in the second subsection. Results of nine numerical experiments are used to ensure the efficiency of the proposed image deconvolution method.

### 4.1. Experiments on the Frequency Blur Invariant

In order to verify the proposed blur invariant in (12), binary and gray-scale images of size 32 × 32 are used. The blurred images are obtained using different variance, *σ* ^2^, by a mask with size of 3 × 3.  Table 1 shows the original binary and its corresponding blurred images. In this table, the blur invariants shown in (12) are denoted as Ψ(*u*, *v*), where the frequencies, *u* and *v*, are varied in random ranges. In each row of this table, the results of the amplitude and phase of the blur invariants are shown. It can be observed that their respective values remain the same or slightly change for different *σ* ^2^.

The results shown in Table 2 for “Saturn” gray-scale image indicate similar observations of blur invariants as in Table 1. One thing to observe for both the tables is that the values of the blur invariants vary slightly with different *σ* ^2^. This is because (8) was based on the integral form whereas all invariant computations are executed in discrete form, which may lead to numerical error in the calculation. The proposed blur-invariant values are fairly stable with respect to different Gaussian kernel, *σ* ^2^.

### 4.2. Experiments on the Proposed Image Deconvolution

To validate the proposed image deconvolution shown in (20), an iterative procedure is performed. Rewriting (20) in terms of iterations, we obtain the estimated restored image as

**Figure 1:**
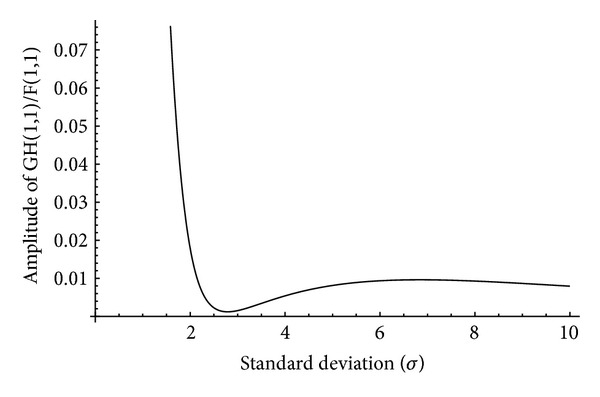
Variation of |*G*(1,1)/*F*(1,1)| with respect to the standard deviation of the Gaussian blur kernel for “Saturn” image shown in Table 2.

**Figure 2:**
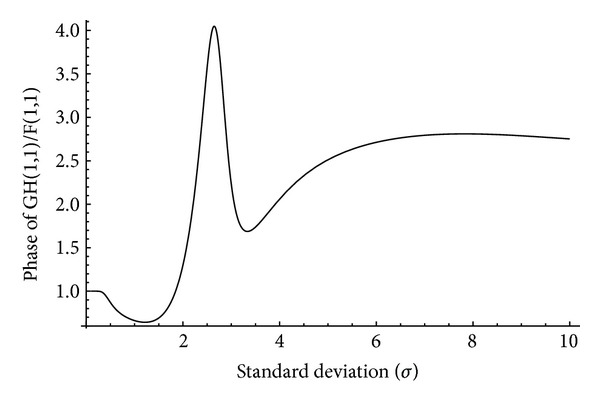
Variation of *∠G*(1,1)/*F*(1,1) with respect to the standard deviation of the Gaussian blur kernel for “Saturn” image shown in Table 2.

(21) fi^(x,y)=∑k=0∞∑l=0∞1k!l!(−σi−122)k+l∑m=02k∑n=02l(−1)2k+2l−m−n  ×(2km)(2ln)g(x−k+m,y−l+n),

where

(22) $$\begin{array}{c} e^{-\sigma_{i}^{2}}=\frac{G\left(1,1\right)}{F{\left\{{\hat{f}}_{i-1}(x,y)\right\}}_{u=v=1}} \end{array}$$

and *i* is the iteration number. In this technique, an estimation of the original image is obtained after every iteration. To estimate the value of *σ* in (11), the only unknown parameter is *F*(1,1) which can be replaced by a suitable Fourier transform of the blurred version of the original image such as *G*(0,0). To understand the behavior of *G*(1,1)/*F*(1,1) in terms of the variation of standard deviation (*σ*), we plot the amplitude and phase of this factor for different amount of blur from 0.1 to 10 for “Saturn” image that is shown in Table 2. Figures 1 and 2 show the variation of amplitude and phase of the original and blurred images' Fourier transform in terms of *σ*. It can be seen that the amplitude of *G*(1,1)/*F*(1,1) is decreasing up to *σ* ≈ 2.75 uniformly, whereas the phase of that is increasing up to the same point of standard deviation.

To measure the improved quality of the restored images, the normalized mean square error (NMSE), ${||\left({\hat{f}}_{i}\left(x,y\right)-{\hat{f}}_{i-1}\left(x,y\right)\right)/{\hat{f}}_{i}\left(x,y\right)||}^{2}$, has been used as a reference metric. The iteration stops once a minimum value of NMSE is reached.

**Figure 3:**
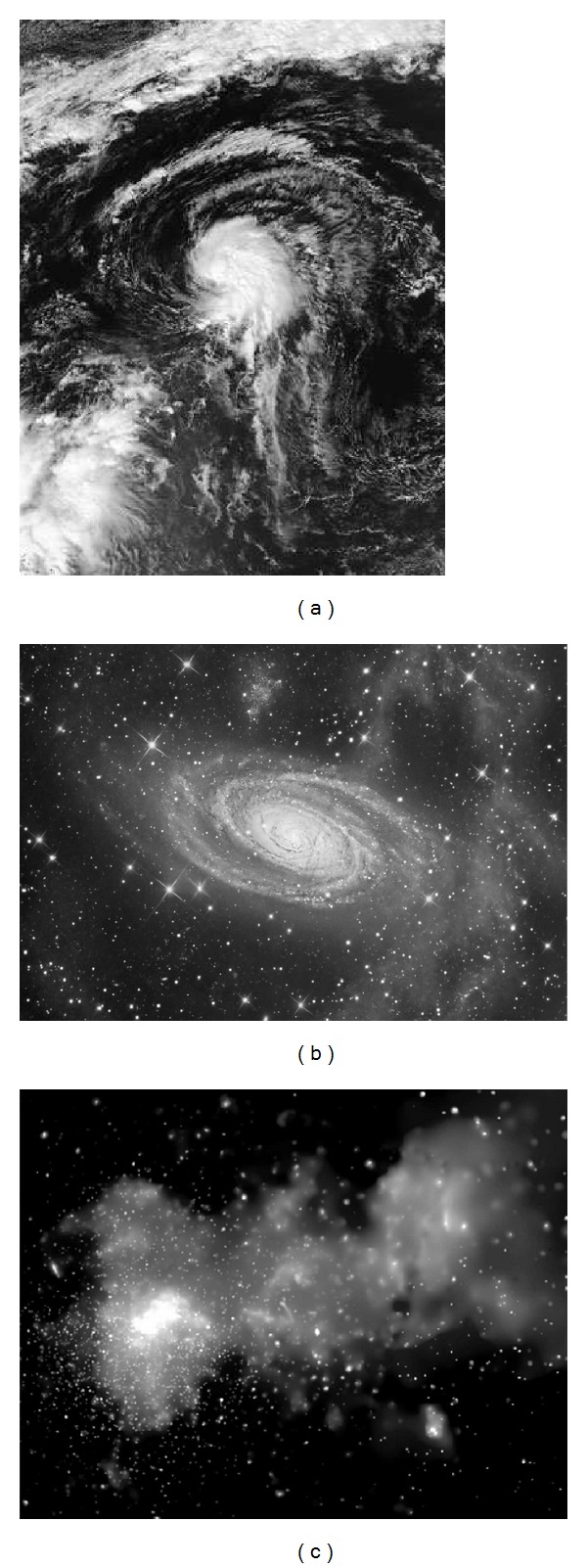
Set of gray-scale images used in the experiments: (a) Tropical Storm Lorenzo (337 × 440), (b) Milky Way (1536 × 1056), and (c) Galaxy (1316 × 1032).

This iterative approach is performed on three real astronomical images—Tropical Storm Lorenzo (Image A) of size 337 × 440, Milky Way (Image B) of size 1536 × 1056, and Galaxy (Image C) of size 1316 × 1032; see Figure 3.

In this experiment, for each of the aforementioned astronomical images, we degraded them using artificial blur by a Gaussian kernel of different mask sizes. Image A is degraded by blur kernel of sizes 5 × 5, 11 × 11, and 17 × 17 with *σ* values of 3.33, 7.66, and 11.33, respectively.  Table 3 illustrates the results for deconvolved images using the proposed frequency blur invariant features. It is clear that, after every step, the quality of the deconvolved image becomes better, and finally, we can get a fine quality of the deconvolved images based on the minimum value of the NMSE. Image B is degraded by blur kernel of sizes 7 × 7, 19 × 19, and 23 × 23 with *σ* values of 4.66, 11.33, and 15.33, respectively. As can be seen from Table 4, the process converges reaching *σ* = 4.94, 11.41, and 14.95. for different mask sizes and yielding visually very good result with small NMSE. Finally, Image C is degraded by blur kernel of sizes 11 × 11, 19 × 19, and 23 × 23 with *σ* values of 7.33, 12.66, and 14.99, respectively. The same deblurring processes are shown in Table 5 for different level of blurs of Galaxy image. One can observe from these three tables that it yields very good results for the overestimating of *σ* values. The advantage of *σ* overestimation can be seen in the first and second rows of Tables 3 and 4 and also in the below row of Table 5.

**Figure 4:**
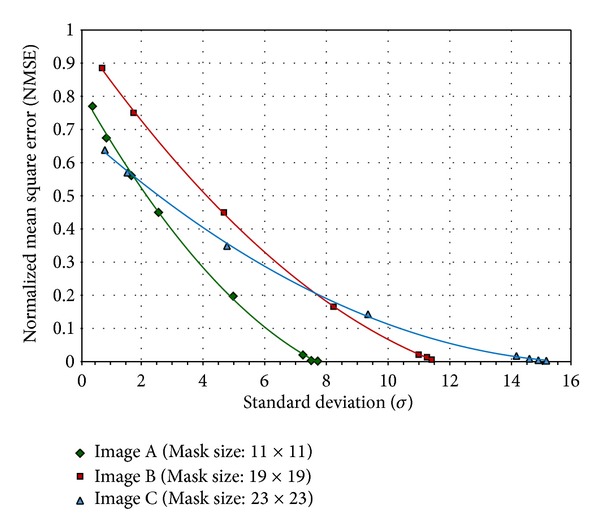
NMSE for three restored images of Table 3.

The plotted curves of NMSE for three images are displayed in Figure 4. It would be noted that the three curves of NMSE are plotted in the same figure in terms of standard deviation of blur kernel for easier comparison. As shown in Figure 4, the NMSE curves of the restored images approach zero by increasing the *σ* values. The results of these experiments ensure the robustness of the proposed Fourier domain blur invariant.

## 5. Conclusion

In summary, we presented a novel blur invariant technique in frequency domain using Fourier transform properties of a Gaussian PSF kernel. The proposed features are equal in both original and blurred images which are described in Fourier domain. To our knowledge, this represents a normalization of the Fourier transform of the original and degraded images by their respective fixed frequencies which are set to one. In addition, the obtained blur invariant features will enable us to estimate the original image which is degraded by a Gaussian kernel. We use this invariant not only to restore the degraded images, but also to evaluate the variance of the PSF. Since the proposed image deblurring algorithm is similar to the nonblind deconvolution, we applied the NMSE factor to show the error measurement and the image quality in these analyses. Finding other types of image quality measurement to determine an appropriate *σ* range for real image deconvolution is a major direction for further practical applications on the proposed method.

## Figures and Tables

**Table 1:** The original binary and its blurred images with the proposed invariants of the blurred images.

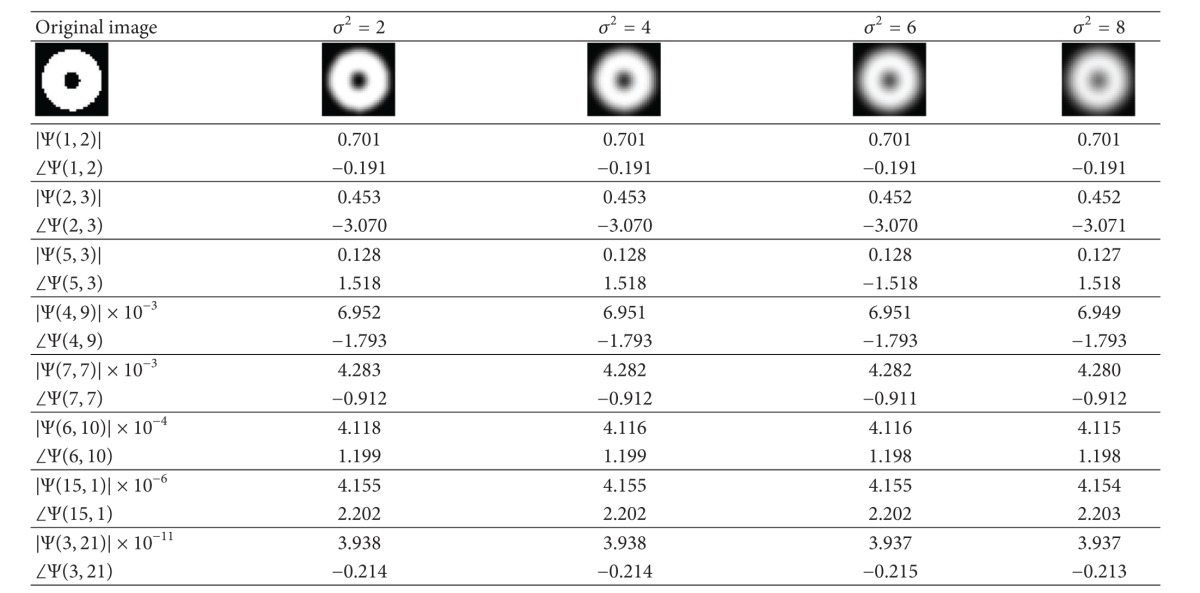

**Table 2:** The original gray-scale and its blurred images (“Saturn” image) with the proposed invariants of the blurred images.

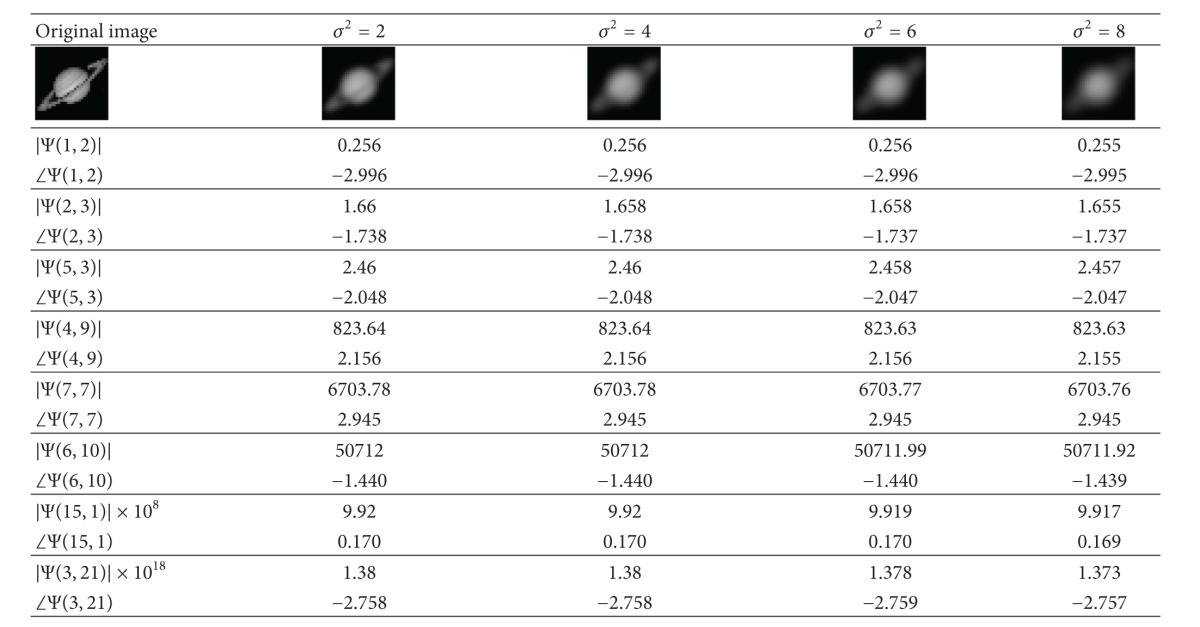

**Table 3:** Image deconvolution using proposed frequency blur invariant for Tropical Storm Lorenzo image (shown in Figure 3(a)) with different estimated *σ* (below the restored images), different mask sizes, and their corresponding NMSE.

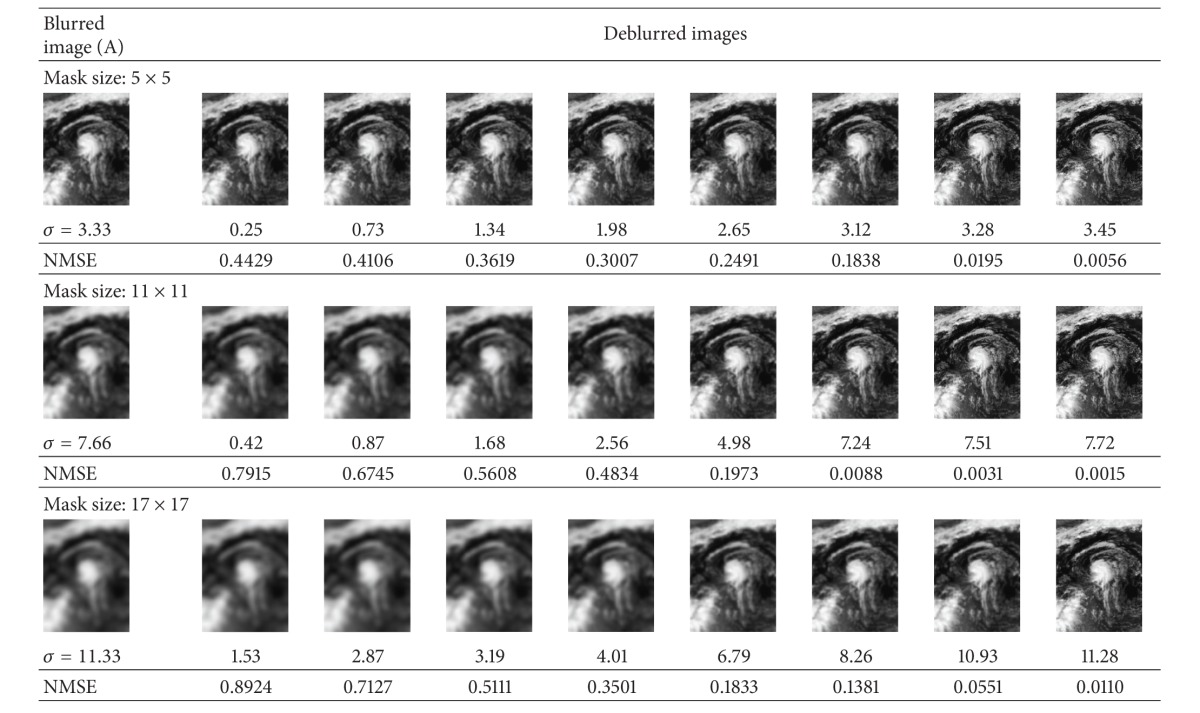

**Table 4:** Image deconvolution using proposed frequency blur invariant for Milky Way image (shown in Figure 3(b)) with different estimated *σ* (below the restored images), different mask sizes, and their corresponding NMSE.

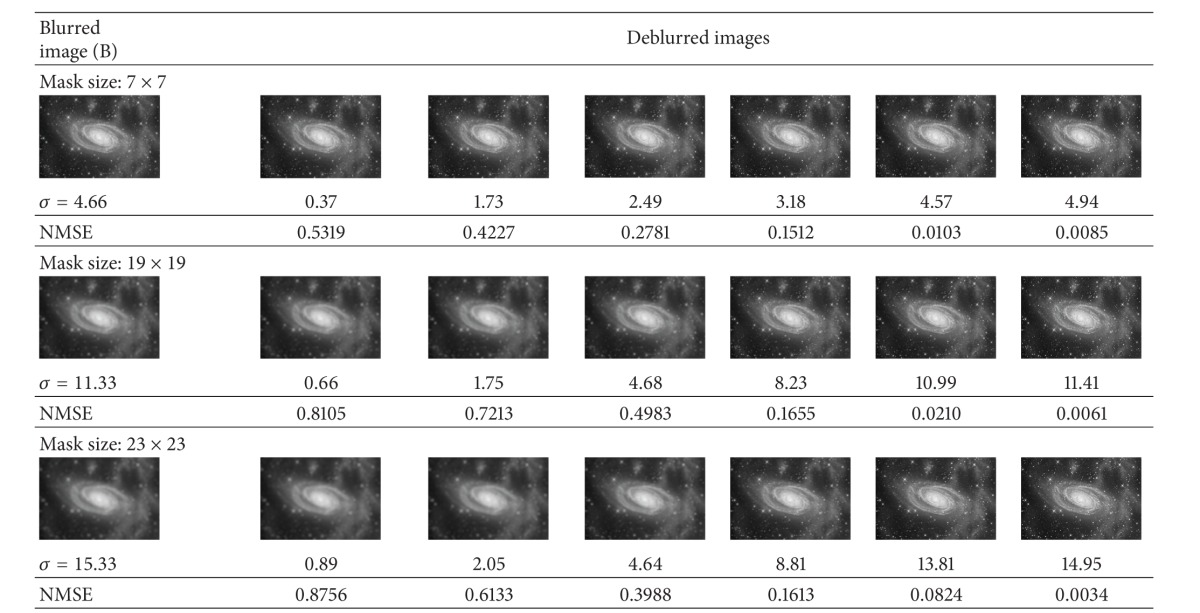

**Table 5:** Image deconvolution using proposed frequency blur invariant for Galaxy image (shown in Figure 3(c)) with different estimated *σ* (below the restored images), different mask sizes, and their corresponding NMSE.